# Prebiotic diet normalizes aberrant immune and behavioral phenotypes in a mouse model of autism spectrum disorder

**DOI:** 10.1038/s41401-024-01268-x

**Published:** 2024-04-08

**Authors:** Naika Prince, Lucia N Peralta Marzal, Anastasia Markidi, Sabbir Ahmed, Youri Adolfs, R Jeroen Pasterkamp, Himanshu Kumar, Guus Roeselers, Johan Garssen, Aletta D Kraneveld, Paula Perez-Pardo

**Affiliations:** 1 Division of Pharmacology, Faculty of Science, Utrecht Institute for Pharmaceutical Sciences, Utrecht University, 3584 CG Utrecht, The Netherlands; 2 Division of Cell Biology, Metabolism & Cancer, Department of Biomolecular Health Sciences, Utrecht University, 3584 CL Utrecht, The Netherlands; 3 Department of Translational Neuroscience, UMC Utrecht Brain Center, University Medical Center Utrecht, Utrecht University, 3584 CG Utrecht, The Netherlands; 4 Danone Nutricia Research, 3584 CT Utrecht, The Netherlands; 5 Department of Neuroscience, Faculty of Science, VU university, 1081 HV Amsterdam, The Netherlands

**Keywords:** autism spectrum disorder, prebiotics, gastrointestinal microbiome, immunomodulation, gut-brain axis

## Abstract

Autism spectrum disorder (ASD) is a cluster of neurodevelopmental disorders characterized by deficits in communication and behavior. Increasing evidence suggests that the microbiota-gut-brain axis and the likely related immune imbalance may play a role in the development of this disorder. Gastrointestinal deficits and gut microbiota dysfunction have been linked to the development or severity of autistic behavior. Therefore, treatments that focus on specific diets may improve gastrointestinal function and aberrant behavior in individuals with ASD. In this study, we investigated whether a diet containing specific prebiotic fibers, namely, 3% galacto-oligosaccharide/fructo-oligosaccharide (GOS/FOS; 9:1), can mitigate the adverse effects of *in utero* exposure to valproic acid (VPA) in mice. Pregnant BALB/cByJ dams were injected with VPA (600 mg/kg, sc.) or phosphate-buffered saline (PBS) on gestational day 11 (G11). Male offspring were divided into four groups: (1) *in utero* PBS-exposed with a control diet, (2) *in utero* PBS-exposed with GOS/FOS diet, (3) *in utero* VPA-exposed with a control diet, and (4) *in utero* VPA-exposed with GOS/FOS diet. Dietary intervention started from birth and continued throughout the duration of the experiment. We showed that the prebiotic diet normalized VPA-induced alterations in male offspring, including restoration of key microbial taxa, intestinal permeability, peripheral immune homeostasis, reduction of neuroinflammation in the cerebellum, and impairments in social behavior and cognition in mice. Overall, our research provides valuable insights into the gut-brain axis involvement in ASD development. In addition, dietary interventions might correct the disbalance in gut microbiota and immune responses and, ultimately, might improve detrimental behavioral outcomes in ASD.

## Introduction

Autism spectrum disorder (ASD) is a group of neurodevelopmental disorders marked by social interaction, communication difficulties and cognitive deficits [1, 2], as well as repetitive or restricted behaviors. Over the past decade, the global prevalence of ASD has increased, and the disorder is four times more likely to occur in boys than in girls [3, 4]. The exact etiology of ASD is not fully understood; however, emerging research indicated a significant interplay between genetic susceptibilities and environmental influences, including accumulating evidence suggesting the potential involvement of the gut-brain in its pathogenesis.

A meta-analysis revealed that children with ASD experience four times more gastrointestinal (GI) problems than controls [5]. Another study reports a correlation between the GI problems and the severity of the behavioral problems [6]. Evidence supporting the involvement of the gut-brain axis includes studies analyzing the gut microbiota composition of individuals with ASD, which have reported alterations in the microbial population [7]. Animal studies support this association as well, with studies demonstrating that microbiota transplantation from individuals with ASD to rodents resulted in the manifestation of autistic-like behaviors [8–11]. Taken together, these findings underscore the microbiome’s critical role in ASD, suggesting a microbiome-based approach as a potential treatment for ASD [12–15].

Prebiotics, non-digestible oligosaccharides that selectively promote the growth of beneficial bacteria and modulate gut microbiota composition, have emerged as a promising novel therapeutic approach for ASD [16, 17]. The fermentation of prebiotics by beneficial bacteria produces short-chain fatty acids (SCFA) among other compounds, which improve intestinal epithelial barrier integrity and function, and promote mucosal immune system homeostasis [18–20]. Additionally, prebiotics have been demonstrated to have beneficial effects on the serotonergic system [21], neuroinflammation [22], and behavior, including cognition and social behavior. These findings indicate that prebiotics can effectively modulate the gut-brain axis by targeting multiple factors that are altered in ASD [23–26]. However, despite the evidence supporting the therapeutic potential of prebiotics, further research is needed as the field is still in its early stages.

This study aimed to examine the potential mechanisms by which gut microbes influence behavioral changes in a mouse model of ASD, and to investigate early prebiotic dietary intervention as a possible treatment strategy. We utilized the valproic acid (VPA) mouse model, which displays a behavioral phenotype similar to ASD and GI abnormalities accompanied by changes in gut microbiota composition [27, 28]. Importantly, only male mice were used in our study, as female mice do not display the ASD-phenotype [27]. Mice were fed a combination of two prebiotics, galacto-oligosaccharide (GOS) and fructo-oligosaccharide (FOS), at an early developmental stage. The choice of prebiotic fibers was based on their established ability to modulate the immune system [29–31], a central factor in the gut-brain axis and implicated in ASD pathology. Specifically, these dietary fibers have been shown to suppress allergic reactions [32], diminish the incidence of infections [33], enhance immune competence [34, 35], and reduce inflammation [36]. Hence, we evaluated the immune system as potential mediator of the effects of prenatal exposure to VPA on the development of ASD symptoms in the male offspring, and as potential target through which prebiotics might modulate the gut-brain axis.

## Materials and methods

### VPA-induced mice model

**Fig. 1:**
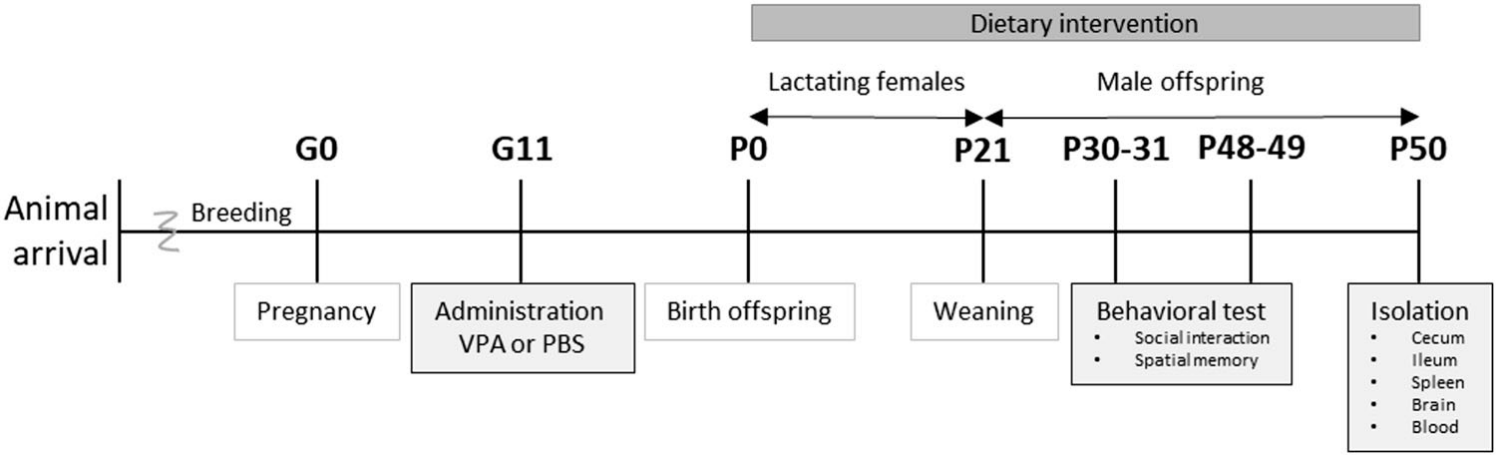
A schematic overview of the experimental design. Mice were exposed prenatally to PBS or VPA on G11. Offspring are exposed to dietary intervention from birth, first trough lactation and after weaning at P21 by GOS/FOS supplemented pellets. Spatial memory evaluation (at P30 and P48) and social interaction test (at P31 and P49) were performed two times throughout the experiment. Finally, on P50, all mice were sacrificed and the cecum, ileum, spleen, blood and brain were collected and adequately stored for further analysis.

Eight-week-old male (*n* = 8) and female (*n* = 16) BALB/cByJ mice were obtained from Charles River Laboratories (Maastricht, The Netherlands) and were housed under standard conditions with a 12 h light/dark cycle, with *ad libitum* access to food and water. A schematic overview of the experimental design is shown in Fig. 1. All females were mated for two consecutive days, and gestational day 0 (G0) was designated when a vaginal plug was present. On G11, dams were injected subcutaneously with 600 mg/kg VPA to induce an autistic-like phenotype in the offspring or PBS as a control. Beginning at postnatal day 0 (P0), lactating dams were provided predetermined diets, which continued until weaning of the pups, who were housed with their mothers, on P21. This dietary regimen was then sustained in the pups post-weaning. Male offspring were subsequently divided into four groups according to exposure and maternal diet. The groups were determined as follows: (1) *in utero* PBS-exposed males fed a control diet (PBS control), (2) *in utero* PBS-exposed males fed a GOS/FOS diet (PBS GOS/FOS), (3) *in utero* VPA-exposed males fed a control diet (VPA control), and (4) *in utero* VPA-exposed males fed a GOS/FOS diet (VPA GOS/FOS). Behavioral tests for spatial memory and social interaction assessments were carried out at P30 and P31, and P48 and P49, to assess prebiotic effects over time. For all behavioral experiments, two cohorts of mice were tested on separate occasions owing to breeding variability, where cohort I consisted of 25 mice and cohort II consisted of 13 mice. The number of animals used for each test is provided in the respective figure legends. Finally, on P50, all mice were sacrificed by decapitation preceding isoflurane anesthesia, and the intestinal tract, spleen, blood, and brain were collected and stored for further analysis.

This study was conducted in accordance with institutional guidelines for the care and use of laboratory animals of the University of Utrecht, and all animal procedures related to the purpose of the research were approved by the local Animal Welfare Body under an Ethical license provided by the national competent authority (Centrale Commissie Dierproeven), securing full compliance with the European Directive 2010/63/EU for the use of animals for scientific purposes.

### Diets

The formulation of the isocaloric diets was carried out by Danone Nutricia Research (Utrecht, Netherlands) and their production by Ssniff Spezialdiäten (Soest, Germany). All processes were performed in accordance with the American Institute of Nutrition Rodent Diets Guide for Growing and Gestating Mice (AIN-93G). Two different diets were developed and used: AIN-93G (Control) and AIN-93G containing 3.0% GOS:FOS (9:1) (GOS/FOS). Based on the amount and structure diversity of non-digestible oligosaccharides in human milk, a 9:1 mixture of GOS/FOS was used [37]. The food was stored at −20 °C until use.

### Spatial memory test

On P30 and P48, cognitive function was assessed using a spatial memory test. As previously described [38, 39], mice were individually subjected to five consecutive 5-min sessions, with an interval of 3 min during which animals were returned to a waiting cage located inside the test room. During session 1 (S1), the mice were introduced to the open field (OF) (45 cm^2^). From S2 to S4, five distinct objects (distinctly shaped and colored wooden blocks) were placed in the OF to allow the mice to familiarize themselves with the spatial configuration of the objects. During S5, the spatial configuration of the objects in the OF was changed by repositioning two objects (displaced objects, DO) while keeping the other three objects unchanged (non-displaced objects, NDO). The ability of the mice to selectively react to spatial changes was analyzed by calculating a discrimination index (DI) as follows:

1 $${DI}= 	 ({mean}\,{time}\,{with}\,{DO}[S5]-{mean}\,{time}\,{with}\,{DO}[S4]) \\ 	 -({mean}\,{time}\,{with}\,{NDO}[S5]-{mean}\,{time}\,{with}\,{NDO}[S4])$$

A contact was defined as the snout of the animal touching an object.

### Social interaction test

On P31 and P49, sociability in each mouse was assessed based on a previous description [27, 40, 41]. Briefly, mice were individually placed in an OF (45 cm^2^), with two small perforated Plexiglas cages (⌀ 10 cm) located opposite to each other, allowing visual, olfactory, and minimal tactile interaction. Mice were habituated to the OF for 5 min. Subsequently, age- and sex-matched healthy unfamiliar interaction mice (BALB/cByJ) were placed in one of the perforated Plexiglas cages and mice were allowed to interact with the novel social target in a second 5-min session. Exploration behavior was defined as sniffing or touching the cage. The time spent with the target mouse or the empty cage was measured. Sociability index (SI) was calculated as follows:

2 $${SI}=\frac{\left({time}\,{with}\,{target}\,{mouse}-{time}\,{with}\,{empty}\,{cage}\,\right)}{\left({time}\,{with}\,{target}\,{mouse}+{time}\,{with}\,{empty}\,{cage}\right)}$$

### Western blot

Cerebellum (CRB) was isolated and stored at −80 °C. CRB tissue (10–35 mg) was transferred into homogenization tubes (Precellys lysing kit P000918-LYSKO-A, Bertin Technologies) containing RIPA buffer (Thermo Scientific, 89901) with Proteinase Inhibitor Cocktail (1:200; Roche, 11836153001). The brain tissues were homogenized using a Precellys 24 homogenizer (Bertin Technologies, Montigny-le-Bretonneux, France) three times for 10 s at 6000 × *g* with a 10 s pause between the homogenization steps. Protein concentrations were determined using Pierce™ BCA Protein Assay Kit (Thermo Scientific, 23225) according to the manufacturer’s instructions. A total of 15 µg protein was loaded onto a 4%–15% gradient precast polyacrylamide gel (Bio-Rad, 5671084), separated by electrophoresis, and transferred onto PVDF membranes (Bio-Rad, 1704157) with a Trans-Blot Turbo Transfer System. The blots were blocked with 5% milk powder in PBST (0.1% Tween 20 in PBS) at room temperature for 2 h. Afterwards, blots were incubated with primary antibodies overnight at 4 °C, followed by peroxidase-conjugated goat anti-rabbit or rabbit anti-mouse secondary antibodies (1:2000, Dako, P0448, and P0260, respectively) at room temperature for 1 h. Immunoproducts were detected by chemiluminescence with Clarity Western ECL Substrate or Clarity Max Western ECL Substrate (Bio-Rad, 1705061 or 1705062) and were imaged using the ChemiDoc MP Imaging System (Bio-Rad Laboratories, Hercules, CA, USA) to detect bands. ImageJ software (version 1.52r, National Institutes of Health, USA) was used to quantify the density of the bands. The following primary antibodies were used: TMEM119 (1:1000, Proteintech, 66948-1; marker of microglial activation) [42], CD68 (1:500, Bio-Rad, MCA341R; marker of activated phagocytic microglia) [43], and GAPDH (1:2000, Cell Signaling, 2118) as loading control.

### iDisco and light sheet imaging

The number of serotonergic neurons and the morphological profile of microglia in mouse brains were determined by immunolabeling-enabled three-dimensional imaging of solvent-cleared organs (iDISCO) [44, 45]. The left hemispheres were pre-treated and cleared as described previously [46]. Samples were immunolabeled using goat anti-Tph2 (1:1000, Everest biotech, EB-11012) and rabbit anti-Iba1 (1:1000, Thermo-fisher, PA5-27436) for serotonergic neurons and microglial cells, respectively. Species-matched Alexa-conjugated donkey antibodies were used at 1:500, namely donkey anti-goat alexa fluor (AF) 647 (Thermo-fisher, A21447) and donkey anti-rabbit AF 594 (Thermo-fisher, R37119). After immunolabeling, samples were cleared and stored in dibenzyl ether. Samples were imaged with a light-sheet microscope (Ultramicroscope II, Lavision BioTec, Bielefeld, Germany) equipped with an Olympus MVX10 zoom body, an Olympus MVPLAPO 2× objective lens, Neo sCMOS camera (Andor) (2560 × 2160 pixels; pixel size: 6.5 μm × 6.5 μm) and Imspector software (LaVision BioTec). The effective magnification was 5.263× (zoom body×objective + dipping lens = 2.5 × 2.1052) and 13.263× (zoom body×objective + dipping lens = 6.3 × 2.1052) to visualize Tph2 staining in the raphe nuclei (RN) and Iba1 staining in the medial prefrontal cortex (mPFC), respectively. Samples were scanned using single-sided illumination, and a step-size of 2.5 μm for the RN and 0.5 μm for the mPFC using the horizontal focusing light sheet scanning method with the optimal number of steps and a sheet NA of 0.074. The following laser filter combinations were used: Coherent OBIS 561–100 LS Laser with a 615/40 filter and Coherent OBIS 647–120 LX with a 676/29 filter.

Image analysis was performed using Bitplane Imaris 9.3.0 software (Oxford Instruments, Abingdon, UK). Tph2^+^ cells were quantified, and their position relative to the origin point (*z* = 0 μm) was determined in the RN using the “Imaris Spots” tool. For the Iba1^+^ cells the “Imaris Surfaces” tool was chosen, to measure their volume, the space they occupy in the mPFC and their number.

### Immunohistochemistry and image analysis

The ileum was isolated, fixed in formalin, and embedded in paraffin. 15 μm-thickness sections were used for the immunohistochemical detection of serotonin-positive (5-HT^+^) cells and tight junction protein zonula-occludens-1 (ZO-1). The sections were then deparaffinized. 5-HT staining required an incubation with 0.3% H_2_O_2_ in methanol for 30 min followed by a rehydration step. Whereas ZO-1 required an incubation step with citrate buffer (0.01 M). Following serum block, ileal sections were incubated overnight at 4 °C with the appropriate primary antibodies: rabbit anti-5-HT (1:8000, Sigma, S5545) and rabbit anti-ZO-1 (1:300, Thermo Fisher Scientific, 40-2200). For 5-HT staining, the ileal sections were washed and incubated with biotinylated goat anti-rabbit secondary antibody (1:200, Dako, P0448). After the sections were washed, the avidin-biotin method was used to amplify the signal (Vectastain ABC Kit; Vector Laboratories, PK-6100), and 3,3’-diaminobenzidine tetrachloride (DAB, Sigma, D4293) was used as chromogen. Sections were counterstained with Mayer’s hematoxylin (Merck Millipore, 109249). For ZO-1 staining, ileal sections were washed and incubated with a fluorescent secondary antibody, goat anti-rabbit AF 594 (1:200, Thermo Fisher Scientific, A-11072) and mounted using ProLong Gold Antifade Mountant with DAPI (Invitrogen, P36931). For 5-HT staining, digital images were acquired using an Olympus BX50 microscope (Tokyo, Japan) equipped with a Leica DFC 320 digital camera at a magnification of 20×. 5-HT^+^ cells in the epithelial layer of the intestinal mucosa were manually counted in six consecutive villi at a minimum of five locations in the intestinal sections of each mouse. Data are expressed as the number of 5-HT positive cells per 10 villi.

For ZO-1, a Keyence BioRevo BZ-9000 microscope (Osaka, Japan) was used for the image acquisition. The lens that was selected had a numerical aperture of 0.5 and a magnification of 20×. The exposure time was maintained at 1.2 s for all images to ensure consistency using a high-pressure mercury lamp. ZO-1 expression levels were subsequently assessed by measuring the Corrected Total Fluorescence (CTF), integrated density – (area × mean fluorescence of background reading) for 10 images per sample using ImageJ software.

### Flow cytometry analysis of T helper cells in the spleen

After collection and homogenization of the spleen (including red blood cell lysis), single-cell suspensions were used to analyze the T helper (Th) cell subsets by flow cytometry. Cells (1 × 10^6^ cells/well) were collected in fluorescence-activated cell sorting (FACS) buffer (PBS containing 1% bovine serum albumin) and plated. The cells were blocked for 20 min with Fc block (anti-mouse CD16/32, Invitrogen, 14-0161-86). Subsequently, cells were stained with the following antibodies in FACS buffer for 30 min at 4 °C: anti-CD4-BV510 (BioLegend, 100553), anti-CD69-PE-Cyanine7 (Invitrogen, 25-0691), anti-CD196-PE (BioLegend, 129803), anti-CD25-PerCP-Cyanine5.5 (Invitrogen, 45-0251), anti-Tbet-AF 647 (BioLegend, 644803), anti-RoryT-AF 647 (BD Biosciences, 562682), and anti-FoxP3-FITC (Invitrogen, 11-5773). Antibody concentrations were individually titrated beforehand and isotype controls were used. Dead cells were excluded using a fixable viability dye eFluor780 (Invitrogen, 65-0865) and aggregated cells were excluded based on forward/sideward scatter properties. Cut-off gates for positivity were established using the fluorescence-minus-one technique. Cells stained for extracellular markers were fixed using Foxp3 Staining Buffer Set (Invitrogen, 00-5523-00), according to the manufacturer’s instructions. The results were collected using a FACS Canto II (BD Biosciences, Franklin Lakes, NJ, USA) and analyzed using Flowlogic software (Inivai Technologies, Mentone, Australia).

### ELISA

Blood samples collected during sectioning were stored as serum at −80 °C until analysis of mouse haptoglobin (HP) (R&D Systems, DY4409) by ELISA. All procedures were performed in accordance with the manufacturer’s instructions. Samples were analyzed in duplicate.

### SCFA analysis

The cecal content was homogenized by vortexing and diluted in cold PBS (1:10). Samples were subsequently centrifuged, the supernatant was collected, and concentrations of acetic, propionic, butyric, isobutyric, valeric, and isovaleric acids were determined as previously described using a Shimadzu GC2010 gas chromatograph (Shimadzu Corporation, Kyoto, Japan) [28], with 2-ethylbutyric acid as an internal standard.

### rRNA gene sequencing

Half of the cecal content was isolated for total DNA extraction (Qiagen Purification Kit, 28104). The total genomic DNA samples were processed for 16S rRNA library preparation and next-generation sequencing. V4 16S rRNA gene sequencing was performed as previously described using 2 × 250 sequencing with a dual barcoding system on an Illumina MiSeq [47].

### Microbiome analysis

16S rRNA data analysis was performed using the Quantitative Insights into Microbial Ecology (QIIME) 1.8 [47]. Briefly, demultiplexed sequencing outputs were then clustered into Operational Taxonomic Units (OTU) with a 97% similarity threshold, and using Greengenes as a reference set to assign taxonomy to each OTU [48]. OTU tables were rarified at the sequencing depth of 1000 sequences/sample.

The alpha and beta diversities of the gut microbiota were compared between the different groups using the Shannon index, which measures species abundance and evenness, and reciprocal Simpson index, which emphasizes the dominance or rarity of species types, for alpha diversity analysis. For beta diversity, principal coordinate analysis was employed utilizing Bray–Curtis dissimilarity, a measure that quantifies the compositional dissimilarity between two different sets of species. These analyses were conducted using Phyloseq (1.20.0) R package [49]. Moreover, for the analyses of microbial composition, linear discriminant analysis effect size (LEfSe) was used to identify taxa that exhibited distinct relative abundances between 1) PBS control and VPA control, and 2) VPA control and VPA GOS/FOS [50]. Analyses were conducted using the Online Galaxy Version 1.0 interface (https://huttenhower.sph.harvard.edu/home; Accessed April 25, 2020). Linear discriminant analysis (LDA) scores were calculated. The alpha value of the Kruskal–Wallis test between classes was set to 0.05 and the threshold for the LDA score function was set to 2.0.

### Statistical analysis

Differences between groups were statistically analyzed with two-way ANOVA [calculating significant effects of exposure (VPA vs. PBS), diet (GOS/FOS vs. control), and interaction (between exposure and diet)], followed by Tukey’s multiple comparisons test using GraphPad Prism software (version 9.4.0). When the data were not normally distributed, they were transformed. This was applied to the Bacteroidetes/Firmicutes (B/F) ratio, percentage of butyric acid, percentage of isovaleric acid, percentage of valeric acid, percentage of Th17 cells, occupancy index, and SI at P31. Outliers were identified and subsequently removed from the statistical analysis using robust regression and outlier removal method. We performed ADONIS test on microbiome distance matrix to quantify the variation explained by defined variables based on 999 permutations using vegan package in R [51]. To reveal associations between SCFA levels and the relative abundance of key taxa, we calculated Spearman’s rank correlation coefficient using corrplot package in R [52]. Data are presented using boxplots and are considered statistically different when *P* < 0.05. The sample sizes were empirically determined based on our previous experience. Analyses were performed using the GraphPad Prism software (version 9.4.0) and R (version 4.2.2).

## Results

### Prebiotic diet rescues disrupted social and spatial memory function in *in utero* VPA-exposed mice

**Fig. 2:**
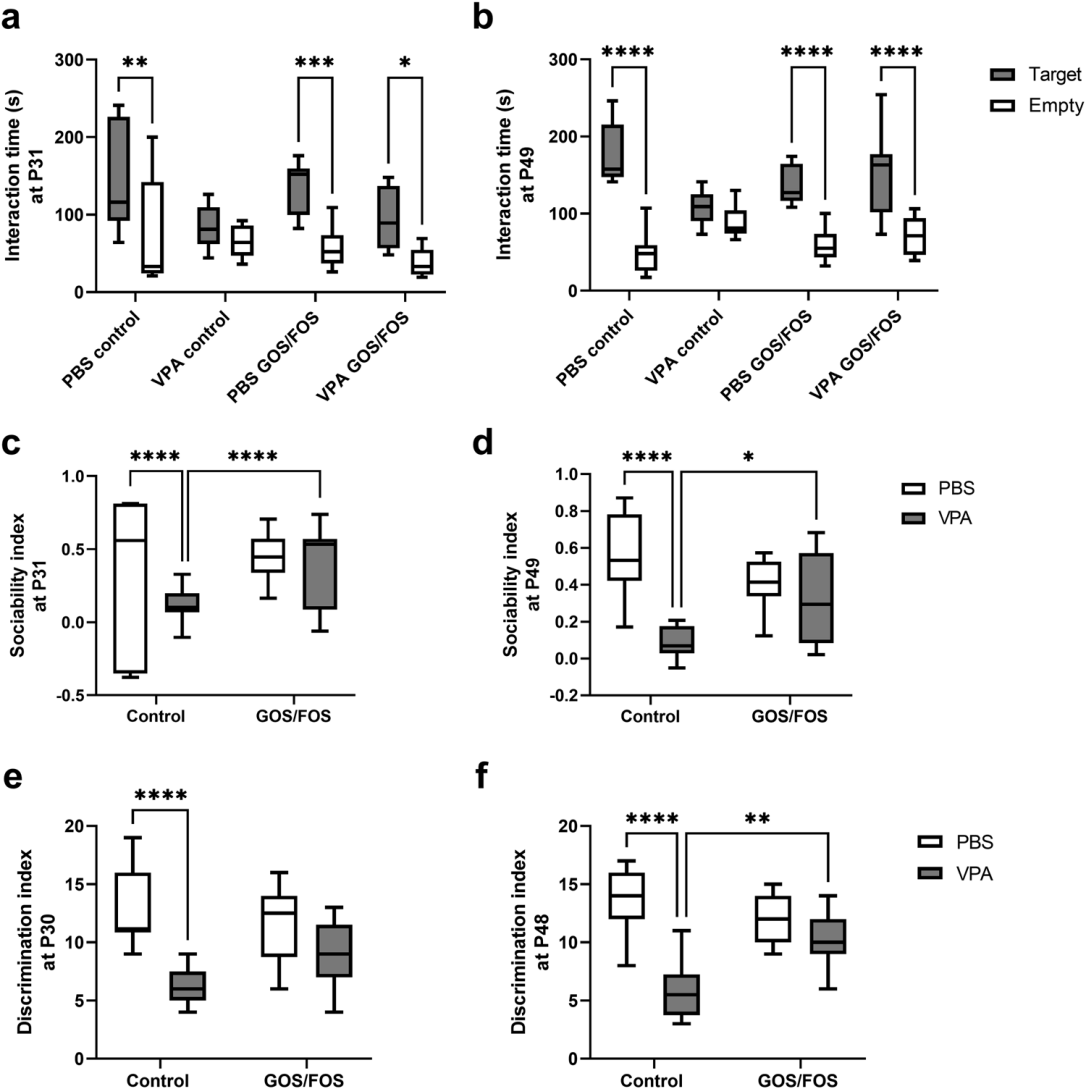
Prebiotic diet rescues disrupted social and spatial memory function in *in utero* VPA-exposed mice. VPA exposure induced social deficits, which were improved by the prebiotic diet at P31 and P49. **a** Time spent interacting with target mouse vs. empty cage at P31, **b** time spent interacting with target mouse vs. empty cage at P49, **c** SI at P31, and **d** SI at P49. Data was presented as time in zone in presence of target mouse. *In utero* PBS-exposed animals selectively re-explored the DO as compared to the NDO. VPA exposure decreased animals’ ability to react to a novel object. VPA GOS/FOS mice showed better spatial discrimination abilities compared to VPA control mice at P48. **e** DI at P30, **f** DI at P48. *P < 0.05, **P < 0.01, ****P < 0.0001, PBS control: *n* = 7–9 mice; VPA control: *n* = 9–10 mice; PBS GOS/FOS: *n* = 10 mice; VPA GOS/FOS: *n* = 9 mice.

To evaluate the impact of the prebiotic diet on behavior, a social interaction test was conducted at P31 and P49. *In utero* PBS-exposed mice spent significantly more time exploring the target mouse rather than empty cage at both P31 (Control: *P* < 0.01, GOS/FOS: *P* < 0.001, Fig. 2a) and P49 (*P* < 0.0001, Fig. 2b), irrespective of the diet. Conversely, VPA control mice showed a lack of social preference (Fig. 2a, b). Remarkably, the GOS/FOS diet completely reversed the sociability deficits of *in utero* exposed VPA mice, as these mice spent significantly more time exploring the target mouse than the empty cage at both P31 (*P* < 0.05, Fig. 2a) and P49 (*P* < 0.0001, Fig. 2b). In addition, *in utero* VPA exposure resulted in a significant reduction of SI compared to *in utero* PBS-exposed offspring at both time points (*P* < 0.0001, Fig. 2c, d), which was significantly increased by GOS/FOS at P31 (*P* < 0.0001, Fig. 2c) as well as at P49 (*P* < 0.05, Fig. 2d). Previous studies have indicated that children with ASD and animal models, in addition to social deficits, have dysfunction in spatial cognition [53–56]. Here, to determine whether VPA exposure could lead to deficiencies in spatial cognition, a spatial memory test was performed at P30 and P48. The *in utero* PBS-exposed animals displayed selective re-exploration of the DO as compared to the NDO throughout the experiment, indicative of their ability to selectively respond to spatial alterations. However, *in utero* VPA exposure impeded the ability of mice to react to spatial modifications compared to PBS control mice at both P30 (*P* < 0.0001, Fig. 2e) and P48 (*P* < 0.0001, Fig. 2f). There was no discernible difference in spatial discrimination ability between VPA control and VPA GOS/FOS mice at P30 (Fig. 2e). Interestingly, at P48, GOS/FOS significantly improved the spatial recognition of *in utero* exposed VPA mice compared with the control diet (*P* < 0.01, Fig. 2f).

### Prebiotic diet resolves phagocytic microglia activation in the CRB of *in utero* VPA-exposed mice

**Fig. 3:**
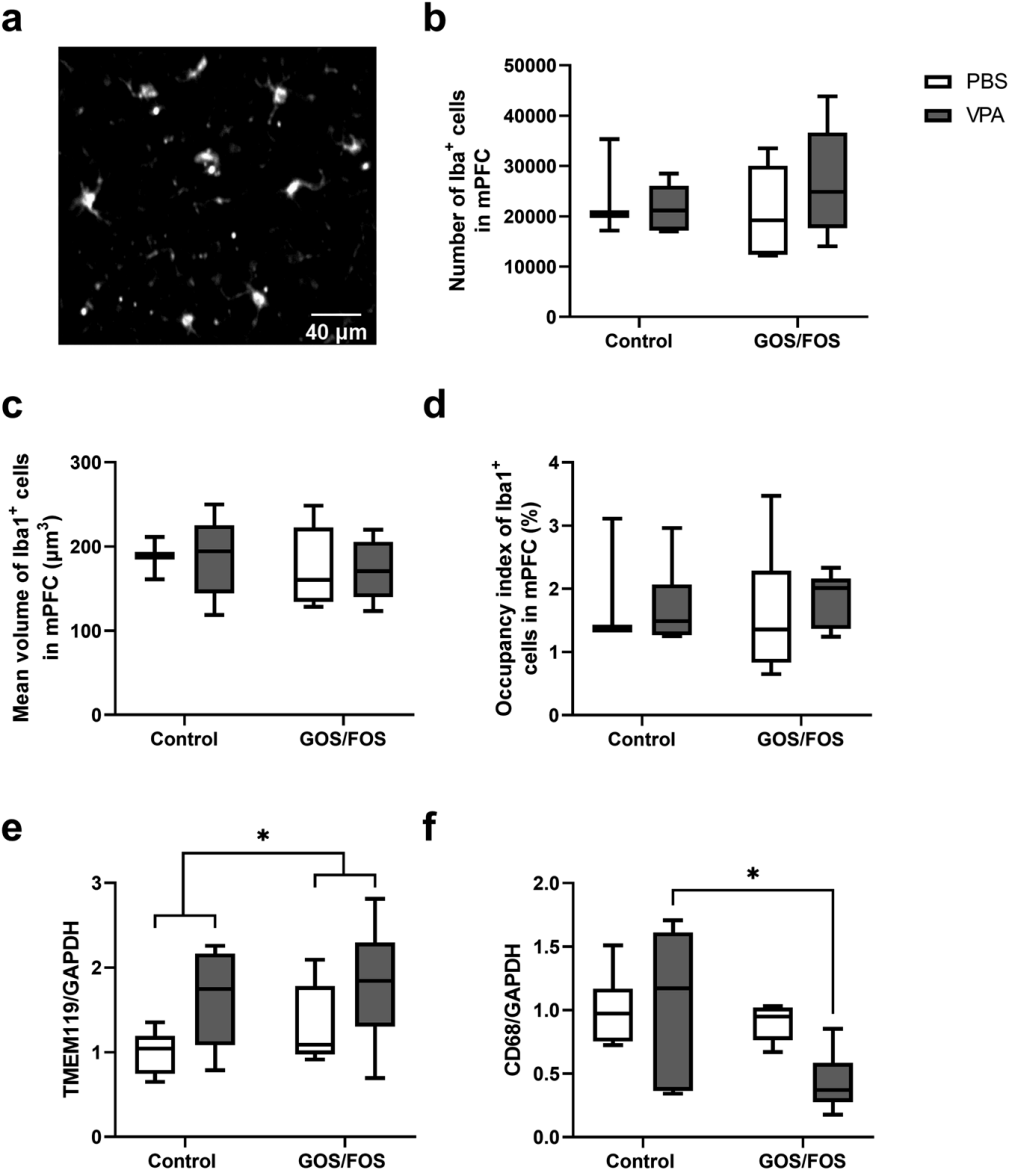
Prebiotic diet resolves phagocytic microglia activation in CRB of *in utero* VPA-exposed mice. Neuroinflammation markers in different brain regions. **a** Representation from mPFC in the control group stained with anti-Iba1, **b** number of Iba1^+^ cells in mPFC, **c** mean volume of Iba1^+^ cells in mPFC, **d** occupancy index of Iba^+^ cells in mPFC, PBS control: *n* = 3 mice; VPA control: *n* = 6 mice; PBS GOS/FOS: *n* = 6 mice; VPA GOS/FOS: *n* = 9 mice. **e** TMEM119 protein expression relative to GAPDH in CRB, and **f** CD68 protein expression relative to GAPDH in CRB. Analyzed blots were processed in parallel. PBS control: *n* = 6 mice; VPA control: *n* = 6 mice; PBS GOS/FOS: *n* = 5 mice; VPA GOS/FOS: *n* = 7 mice. *P < 0.05.

In individuals with ASD, microglia are activated in different brain regions, including the PFC and CRB [57]. To assess the presence and degree of neuroinflammation, the number, volume, and occupancy index Iba1^+^ cells were determined in the PFC, a brain area involved in social behavior and cognition (Fig. 3a-d). No significant effects were found for either *in utero* VPA exposure or diet. In addition to the mPFC, the CRB plays a critical role in various cognitive processes, including working memory, spatial cognition [58], and social behavior [59]. To investigate neuroinflammation in this region, the protein expression levels of TMEM119 (Fig. 3e) and CD68 (Fig. 3f) were evaluated using Western blotting. Our results showed that there was a general effect of *in utero* VPA exposure on TMEM119 expression, which was significantly increased [*F*(1,20) = 5.917, *P* > 0.05] in the CRB compared to control mice, irrespective of the diet [*F*(1,20) = 0.9675, *P* = 0.33], indicating a shift towards a state of microglial activation, a hallmark of neuroinflammation (Fig. 3e). Additionally, the GOS/FOS diet significantly reduced CD68 expression (activated phagocytic microglia) regardless of *in utero* exposure [*F*(1,20) = 5.646, *P* > 0.05], with a significant decrease in CD68 expression observed in VPA GOS/FOS mice compared with to its control (*P* < 0.05, Fig. 3f).

### *In utero* VPA-induced serotonergic cell loss is reduced by the prebiotic diet

**Fig. 4:**
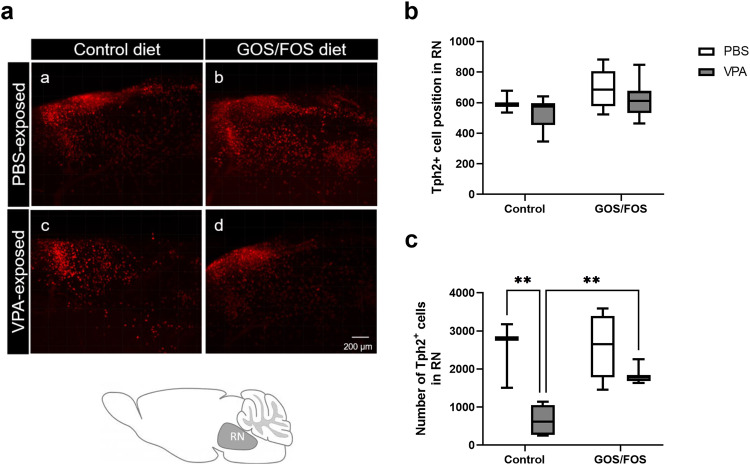
*In utero* VPA-induced serotonergic cell loss is reduced by the prebiotic. **a** (a–d) Representative pictures of Tph2^+^ cells (red) in the RN for each study group, including brain schematic of RN localization, **b** mean distance from a predetermine point of Tph2^+^ neurons through rostro-caudal direction encompassing all subdivisions of the RN. There was not significant change in serotonergic neuron positions. **c** A significant reduction in the number of Tph2^+^ cells was found in VPA-exposed mice compared to PBS-exposed mice fed with control diet. VPA-exposed animals fed with prebiotic diet had normal numbers of Tph2^+^ neurons; **P  <  0.01, PBS control: *n* = 3 mice; VPA control: *n* = 6 mice; PBS GOS/FOS: *n* = 6 mice; VPA GOS/FOS: *n* = 8 mice.

Changes in the serotonergic system associated with behavioral deficits, specifically regarding migration pattern and cell number, are frequently observed in both individuals with ASD and in rodent models of ASD [60–63]. Therefore, the position of serotonergic cells in the RN was first assessed to evaluate any alterations in their migration within the rostro-caudal axis during brain development. The results revealed no influence of *in utero* VPA exposure on the distribution of the cells in the RN [*F*(1,20) = 1.992, *P* = 0.174, Fig. 4b]. Furthermore, the number of Tph2^+^ neurons in the RN was also evaluated to assess impairments in the serotonergic circuit. A significant overall effect of *in utero* VPA exposure was detected on the number of Tph2^+^ cells [*F*(1,20) = 24.31, *P* < 0.0001, Fig. 4a, c]. Specifically, *in utero* VPA exposure resulted in a severe reduction in the number of Tph2^+^ cells compared with that in the PBS control (*P* < 0.01). The prebiotic GOS/FOS appears to significantly mitigate the effect of VPA exposure on the number of serotonergic cells (*P* < 0.01, Fig. 4a:c, d and c:c, d).

### Prebiotic diet reverses the effect of *in utero* VPA exposure on the pro-inflammatory T cell subset population in the spleen and on haptoglobin in serum

**Fig. 5:**
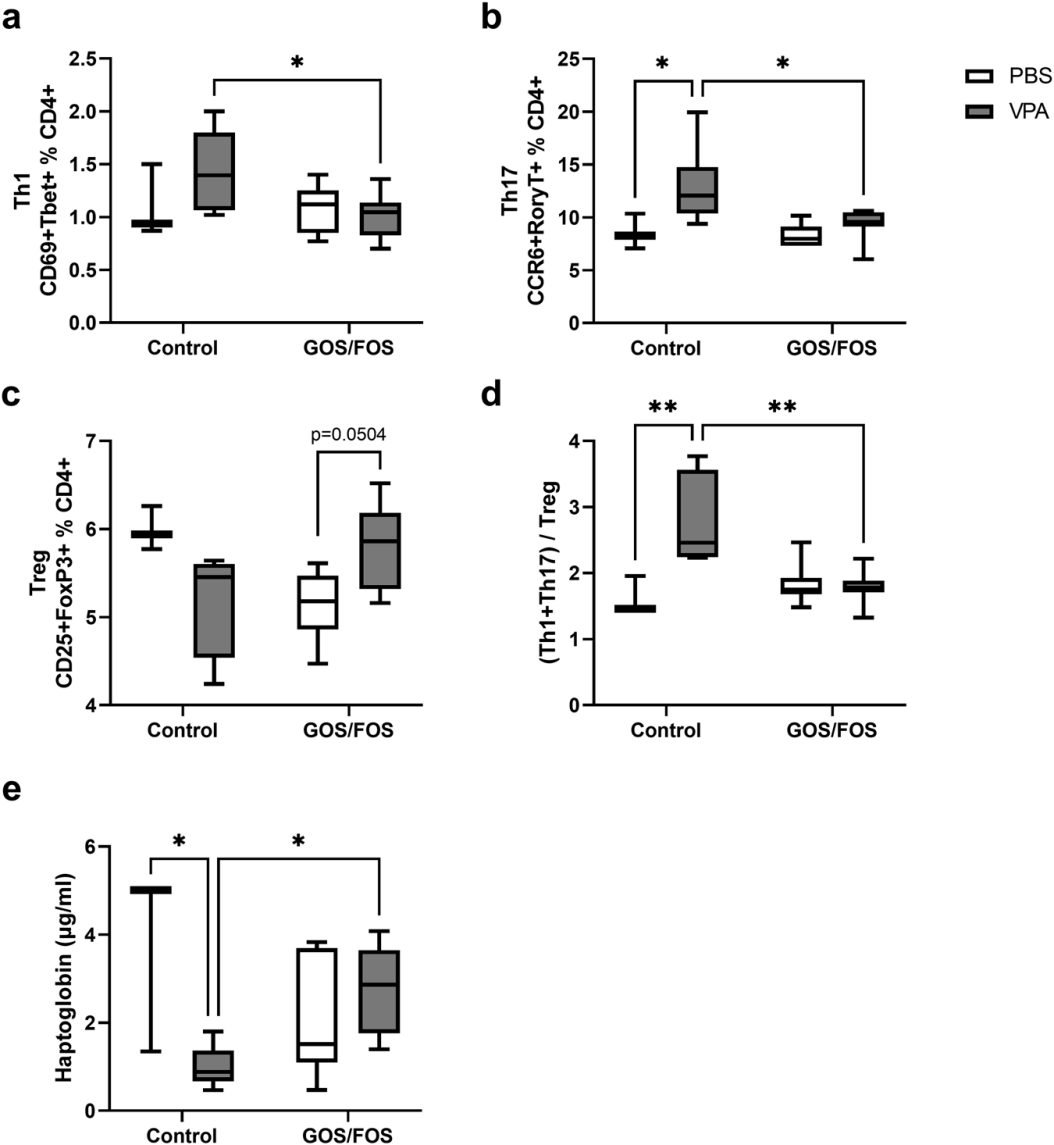
Prebiotic diet reverses *in utero* VPA effects on pro-inflammatory T splenocytes and HP in serum. Flow cytometry analysis of Th cell populations in the spleen at the end of the experimental set up, **a** percentage of activated Th1 cells, **b** percentage of Th17 cells, **c** percentage of Treg cells, **d** ratio of (Th1 + Th17)/Treg cells, **e** HP concentration (µg/ml) in serum. *P < 0.05, **P < 0.01, PBS control: *n* = 3 mice; VPA control: *n* = 6 mice; PBS GOS/FOS: *n* = 7 mice; VPA GOS/FOS: *n* = 8-9 mice.

In the present study, besides investigating microglial activation, we assessed the peripheral immune cell balance to explore the potential link between the gut-brain axis and the immune system regarding ASD. Specifically, the impact of *in utero* VPA treatment and a prebiotic diet on T helper (Th) cell populations were assessed through flow cytometry analysis of splenocytes isolated from mice (Fig. 5). The results revealed no significant difference in the proportion of Th1 cells in the spleens of VPA control mice compared to PBS control mice (*P* = 0.35, Fig. 5a). The GOS/FOS diet significantly decreased the percentage of Th1 splenocytes in *in utero* VPA-exposed mice compared to the control diet (*P* < 0.05). An overall significant effect of VPA exposure was observed on the Th17 cell population [*F*(1,20) = 7.79, *P* < 0.05]. Furthermore, *post hoc* analysis showed a significant increase in the percentage of splenic Th17 cells in VPA control mice compared to that in PBS control mice (*P* < 0.05). This increase was significantly reduced by the GOS/FOS diet in the VPA-exposed mice (*P* < 0.05, Fig. 5b). The proportion of Treg cells was not affected by either VPA exposure or diet, although there was a significant interaction effect between *in utero* exposure and diet [*F*(1,20) = 13.46, *P* < 0.01, Fig. 5c]. In addition, there was a trend towards an increase in the Treg cell population in VPA GOS/FOS mice compared to PBS GOS/FOS mice (*P* = 0.0504). A general effect of *in utero* exposure was observed in the ratio of Th1 and Th17 cells to Treg cells [*F*(1,20) = 9.05, *P* < 0.01]. Moreover, multiple comparison analysis highlighted a higher ratio in VPA control mice than in PBS control mice (*P* < 0.01), which was significantly dampened by the GOS/FOS diet (*P* < 0.01, Fig. 5d).

In addition, the immunomodulatory protein, haptoglobin (HP), was assessed in serum samples (Fig. 5e). A significant interaction effect between VPA exposure and prebiotic diets was observed [*F*(1,21) = 12.84, *P* < 0.01]. Furthermore, *in utero* exposure to VPA significantly reduced serum HP levels (*P* < 0.05), which was reversed by supplementation with a prebiotic diet containing GOS/FOS (*P* < 0.05).

### Prebiotic diet normalizes decreased enterochromaffin cell number and impaired intestinal permeability after *in utero* VPA exposure

**Fig. 6:**
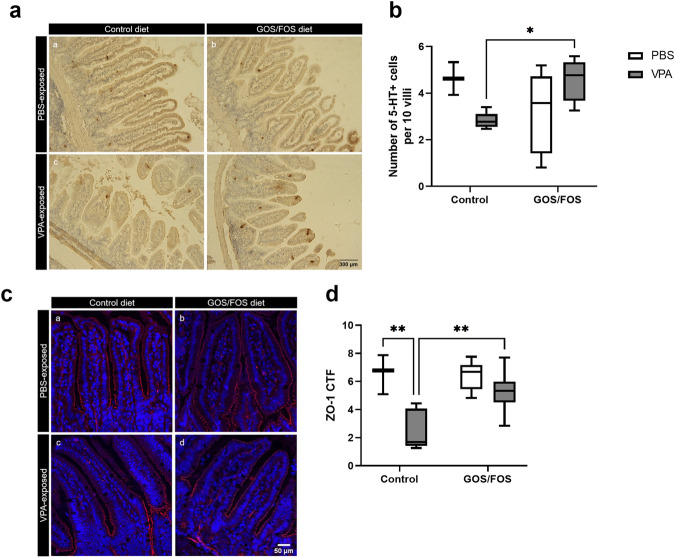
Prebiotic diet normalizes enterochromaffin cell number and intestinal permeability after *in utero* VPA exposure. **a** (a–d) Representative pictures of 5-HT stained ileal sections for each study group, **b** number of 5-HT^+^ cells per 10 villi, **c** (a–d) representative pictures of ZO-1 stained ileal sections for each study group (ZO-1 in red; DAPI in blue), and **d** ZO-1 CTF in the distal ileum of PBS- and VPA-exposed mice fed a control diet and prebiotic diet. *P < 0.05, **P < 0.01, PBS control: *n* = 3 mice; VPA control: *n* = 5-6 mice; PBS GOS/FOS: *n* = 6 mice; VPA GOS/FOS: *n* = 8-9 mice.

Immunohistochemistry was used to detect enterochromaffin (EC) cells in the distal ileum of the mice. These cells are responsible for producing around 90% of 5-HT [64]. Disturbances in the intestinal serotonergic system have been shown to strongly correlate with an impaired serotonergic phenotype in the brain [27]. Results revealed a significant interaction effect between *in-utero* treatment and diet [*F*(1,18) = 8.90, *P* < 0.01]. Specifically, VPA exposure had no significant effect compared to PBS control mice (*P* = 0.21), whereas a significant increase in the number of 5-HT^+^ cells per 10 villi in the epithelial cell layer was observed in VPA GOS/FOS mice compared to VPA control mice (*P* < 0.05, Fig. 6a:c–d and b).

The impact of a prebiotic diet on the integrity of the intestinal epithelium was evaluated by analyzing ZO-1 expression in the distal ileum using immunofluorescence (Fig. 6c:a–d and d). The results indicated an overall effect of VPA exposure [*F*(1,19) = 19.96, *P* < 0.001) and diet [*F*(1,19) = 4.97, *P* < 0.05] on ZO-1 expression in the ileal mucosa. Furthermore, *post hoc* analysis revealed that VPA control mice displayed significantly reduced levels of ZO-1 protein in the ileum compared to PBS-exposed mice (*P* < 0.001, Fig. 6c:a, c, and d). Moreover, the GOS/FOS diet induced a higher expression of tight junction protein in the ileum of *in utero* VPA-exposed mice than the control diet (Fig. 6c:c, d and d, *P* < 0.01), indicating that the GOS/FOS diet restored the disrupted intestinal barrier integrity in VPA mice.

### Prebiotic diet changes bacterial community diversity and composition in cecum and restores key phylotypes

**Fig. 7:**
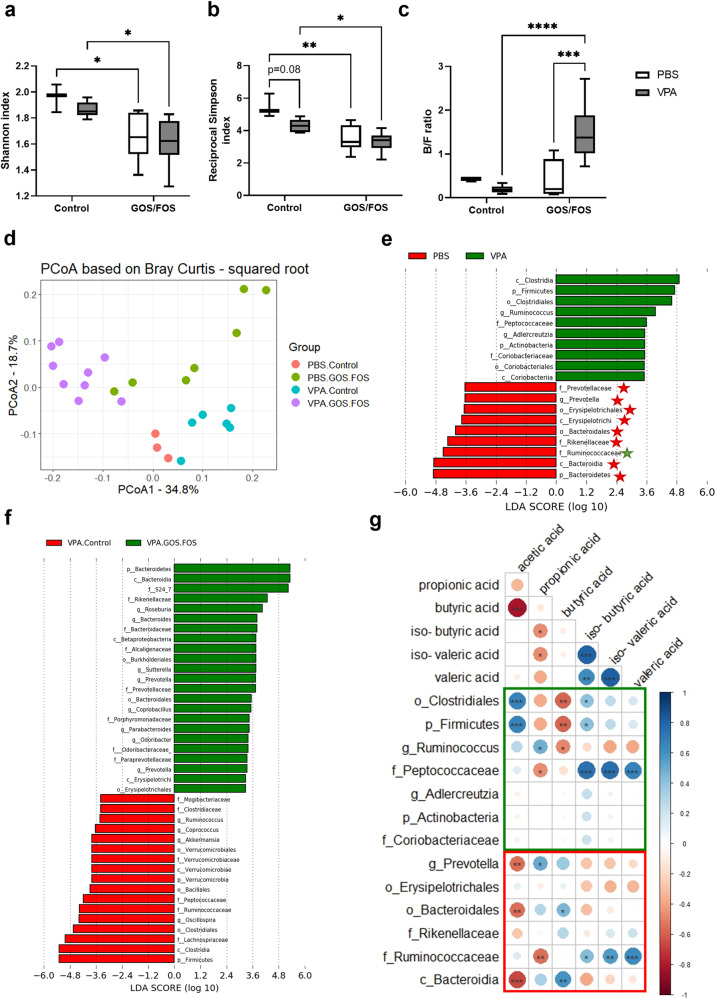
Prebiotic diet changes cecal bacterial diversity and restores key phylotypes in *in utero* VPA-exposed mice. Alpha- and beta-diversity of the gut microbes of PBS- and VPA-exposed mice treated with prebiotics and control diet. **a** Shannon index, **b** reciprocal Simpson index, **c** B/F ratio, **d** PCoA based on Bray–Curtis dissimilarity index, **e** histogram of LDA scores of PBS control (red) and VPA control (green) mice with a cutoff value of LDA score (log_10_) > 2.0 (*P* < 0.05). The stars denote enriched taxa in the VPA GOS/FOS mice. Red and green stars indicate taxa in control and VPA-exposed GOS/FOS mice, respectively. **f** Histogram of LDA scores of VPA control (red) and VPA GOS/FOS (green) mice with a cutoff value of LDA score (log_10_) > 2.0 (*P* < 0.05). **g** Spearman’s correlation between SCFA, and differential bacteria between PBS- (red square) and VPA (green square)-exposed mice determined by LEfSe. Gradient bar refers to rho Spearman values. *P < 0.05, **P < 0.01, ***P < 0.001, ****P < 0.0001, PBS control: *n* = 3 mice; VPA control: *n* = 6 mice; PBS GOS/FOS: *n* = 7 mice; VPA GOS/FOS: *n* = 9 mice.

Bacterial community diversity was measured using both the Shannon index and the reciprocal Simpson index. The results indicated that there was no general effect of VPA exposure on the Shannon index [*F*(1,20) = 1.07, *P* = 0.313, Fig. 7a], while the reciprocal Simpson index was significantly affected by VPA exposure [*F*(1,20) = 6.52, *P* < 0.05, Fig. 7b]. *Post hoc* analysis did not show any statistical differences between the PBS and VPA groups. In addition, the results showed that diet had an overall effect on both indices [*F*(1,20) = 16.72, *P* < 0.001, Fig. 7a] and [*F*(1,20) = 26.72, *P* < 0.0001, Fig. 7b]. Specifically, the GOS/FOS diet significantly reduced the Shannon index when comparing PBS control to PBS GOS/FOS (*P* < 0.05, Fig. 7a) and VPA control to VPA GOS/FOS (*P* < 0.05, Fig. 7a). In addition, *post hoc* analysis showed a significant decrease in the reciprocal Simpson index when comparing PBS control to PBS GOS/FOS (*P* < 0.01, Fig. 7b) and VPA control to VPA GOS/FOS (*P* < 0.05, Fig. 7b). This reduction in diversity reflects an uneven distribution of the microbial community as a result of the GOS/FOS diet, irrespective of *in utero* exposure.

Furthermore, the Bacteroidetes/Firmicutes (B/F) ratio was evaluated (Fig. 7c). There was a significant general effect of *in utero* VPA exposure [*F*(1,21) = 2.466, *P* < 0.05], as well as an interaction effect between *in utero* exposure and diet [*F*(1,21) = 20.12, *P* < 0.001] on the B/F ratio. *Post hoc* analysis showed that VPA exposure did not change the B/F ratio with the control diet (*P* = 0.88). However, the B/F ratio was significantly increased when comparing PBS GOS/FOS mice to VPA GOS/FOS mice (*P* < 0.001). Interestingly, the GOS/FOS diet did not affect the B/F ratio in *in utero* PBS-exposed mice (*P* > 0.99), whereas the GOS/FOS diet significantly increased this ratio in *in utero* VPA-exposed mice (*P* < 0.0001).

Finally, we compared the microbial community structure (beta diversity) among the four populations using the Bray–Curtis dissimilarity matrix (Fig. 7d). The ADONIS test was used to differentiate overall differences in microbiome structure. The microbial community structure between samples showed significant clustering differences between groups (ADONIS *R*^*2*^ = 0.5354, *P* > 0.001), indicating distinct microbial compositions associated with each group. Additionally, the microbial composition was highly influenced by the diet (ADONIS *R*^*2*^ = 0.2421, *P* > 0.001).

To assess taxonomic differences between PBS and VPA exposure in cecal samples, key phylotypes of differentially abundant taxa were identified using LEfSe algorithm at the genus level. The results revealed differences in taxa between *in utero* PBS- (in red) and VPA- (in green) exposed mice (Fig. 7e). Interestingly, the prebiotic diet normalized key bacteria when mice were exposed to VPA (Fig. 7e, f). Of the nine characteristic bacteria in control mice, eight were restored in *in utero* VPA-exposed mice by the prebiotic diet (red stars), regardless of the taxonomic rank, and only *Ruminococcaceae* was not recovered (green star) (Fig. 7e, f). Furthermore, when considering the taxonomic rank more specifically, our analysis revealed that out of six bacteria identified at the lowest taxonomic levels, five were effectively restored in the VPA-exposed mice on the prebiotic diet.

### Correlation between differential taxa and SCFA levels in cecum

To quantify bacterial metabolic activity, the cecal concentrations of SCFA, including acetic acid, propionic acid, butyric acid, isobutyric acid, isovaleric acid, and valeric acid, were measured (Supplementary Fig. S1). The results indicated a significant general effect of *in utero* exposure to VPA on acetic acid [*F*(1,21) = 7.32, *P* < 0.05], propionic acid [*F*(1,20) = 12.07, *P* < 0.01], isobutyric acid [*F*(1,21) = 9.88, *P* < 0.01], and valeric acid levels [*F*(1,21) = 7.389, *P* < 0.05], while the levels of butyric acid and isovaleric acid were unaffected (Supplementary Fig. S1a–f). Conversely, administration of GOS/FOS led to a significant overall effect on propionic acid [*F*(1,20) = 23.68, *P* < 0.0001], isobutyric acid [*F*(1,21) = 36.4, *P* < 0.0001], isovaleric acid [*F*(1,21) = 28.39, *P* < 0.0001], and valeric acid [*F*(1,21) = 42.18, *P* < 0.0001] levels (Supplementary Fig. S1b, d–f). Furthermore, *post hoc* analysis showed no significant differences between PBS- and VPA-exposed mice with the control diet across all SCFA. However, when treated with the GOS/FOS diet *in utero* exposure to VPA significantly decreased acetic acid levels (*P* < 0.01), while significantly increasing levels of propionic acid (*P* < 0.01), isovaleric acid (*P* < 0.001), and valeric acid (*P* < 0.01) (Supplementary Fig. S1a, b, e, f). Additionally, propionic acid levels were significantly higher in VPA GOS/FOS mice compared to VPA control *(P* < 0.0001) but were not changed when comparing PBS groups (Supplementary Fig. S1b). However, isobutyric acid and valeric acid levels were reduced regardless of *in utero* exposure to PBS (*P* < 0.05 and *P* < 0.001, respectively) or VPA (*P* < 0.0001 and *P* < 0.001, respectively) (Supplementary Fig. S1d, e).

To investigate the relationship between microbial activity and the differential taxa determined by LEfSe in Fig. 7e, only using the lowest taxonomic rank, Spearman’s correlations were performed between SCFA levels and differential bacteria (Fig. 7g). A positive correlation was observed between propionic acid and *Prevotella* (*r* = 0.51, *P* < 0.05), and between butyric acid and Bacteroidales (*r* = 0.46, *P* < 0.05). A negative correlation was observed between acetic acid and *Prevotella* (*r* = −0.56, *P* < 0.01). Moreover, a negative correlation was observed between butyric acid and *Ruminococcus* (*r* = −0.49, *P* < 0.05). Whereas acetic acid was positively correlated with Clostridiales (*r* = 0.67, *P* < 0.001). Interestingly, *Peptococcaceae* was positively correlated with the three branched SCFA, isobutyric acid (*r* = 0.75, *P* < 0.001), isovaleric acid (*r* = 0.75, *P* < 0.001), and valeric acid (*r* = 0.66, *P* < 0.001). Lastly, *Ruminococcaceae*, which was not rescued by the prebiotic diet, was negatively correlated with propionic acid (*r* = −0.56, *P* < 0.01), and positively correlated with isobutyric acid (*r* = 0.43, *P* < 0.05), isovaleric acid (*r* = 0.56, *P* < 0.01), and valeric acid (*r* = 0.63, *P* < 0.001). Overall, key taxa found in PBS control mice were positively correlated with butyric acid and propionic acid levels and negatively correlated with isobutyric acid, isovaleric acid, and valeric acid levels, whereas the opposite phenomenon was observed with key phylotypes found in VPA control mice.

## Discussion

The present study aimed to evaluate the potential beneficial impact of specific prebiotic supplementation on autistic-like symptoms in a VPA mouse model of ASD. We hypothesized that supplementing the diet of *in utero* VPA-exposed offspring would modify the composition of the gut microbiota, leading to improved health outcomes. The results indicated that prebiotic supplementation significantly ameliorated ASD-like behavior in in utero VPA-exposed offspring, associated with restored immune homeostasis, improved gut barrier permeability, and restored key bacterial taxa.

*In utero* VPA-exposed male mice typically exhibit social deficits similar to the core symptoms of individuals with ASD [27]. In our study, the GOS/FOS diet reversed these sociability impairments induced by VPA exposure, suggesting its potential as a nutritional approach for addressing behavioral ASD symptoms. Along with improved social abilities, spatial memory deficits were also completely normalized by the prebiotic diet in *in utero* VPA-exposed mice. To the best of our knowledge, this is the first demonstration of such an effect in an animal model of ASD. Similar cognitive improvements have been observed in other prebiotic intervention studies with different neurological conditions [29, 65, 66]. Additionally, beneficial effects on social deficits were observed at an early stage, while the positive impact on spatial working memory required more time. This observation emphasized the critical role of intervention duration, suggesting that a sufficient period of dietary intervention is indispensable to fully realize its positive impact on cognitive-behavioral outcomes. The scope of our study focused on the observation of ASD-like behaviors in male mice exposed to VPA, a phenomenon not observed in females [27]. Although females can be affected by ASD, their symptoms manifest differently compared to their male counterparts [67]. This difference in symptom presentation across sex, observed both in humans and in the VPA mouse model, limits the generalizability of the model. Yet, this also underscores the disorder’s complexity and heterogeneity. Additionally, while administration of VPA during pregnancy is associated with an elevated risk of ASD in children, it is noteworthy that not all exposed individuals manifest autistic traits [68]. Consequently, the utilization of a VPA-induced model inherently encompasses limitations in its ability to fully replicate the human condition. Nevertheless, this model share some common pathways with other etiological factors that contribute to ASD development. Besides limitations inherent to the model [69–72], we recognize the absence of repetitive behavior assessments, one of the core behavioral symptoms of ASD, as a limitation. This exclusion aimed to minimize the testing burden on the mice to prevent stress-related confounding factors that could compromise the reliability of the results. Acknowledging this limitation, we suggest that future research should include a thorough assessment of repetitive behaviors. Despite this limitation, our results offer valuable insights into how prebiotics can influence social behavior and cognitive functions over time. These findings mark a step forward in exploring nutritional strategies for the treatment and management of ASD.

Although the GOS/FOS diet did not affect VPA exposure-induced microglial activation in the CRB, it did significantly decrease CD68 expression. This is in line with a previous study showing that GOS decreased elevated CD68 mRNA levels in the hippocampus after abdominal surgery, which resulted in an improvement of cognitive dysfunction and neuroinflammation [73]. In contrast, no differences were observed in microglial activation in the mPFC, consistent with other studies using the VPA mouse model [27, 74], suggesting a brain region dependency. Overall, the GOS/FOS diet demonstrated an immunomodulatory effect in the brain in the presence of neuroinflammation, mitigating the detrimental effects of *in utero* VPA exposure on social behavior and spatial memory.

Furthermore, *in utero* VPA exposure resulted in serotonergic cell loss in the RN. This is in line with previous studies, which demonstrated that a decrease in serotonergic neurons could lead to a reduction in 5-HT availability and impaired signaling in key brain regions involved in cognition and social behavior, such as the PFC, hippocampus, and CRB, thereby contributing to behavioral abnormalities [75, 76]. Hypothetically, this reduction in neuronal cells may result from the presence of activated microglia in the CRB damaging synaptic connections [77–79]. Therefore, decreasing activated phagocytic microglia, which are responsible for neuronal cell death [77], through dietary intervention might prevent the loss of serotonergic cells and preserve 5-HT signaling in the brain. However, additional studies are needed to confirm the changes in 5-HT neurotransmission by measuring 5-HT concentrations in relevant brain regions. Nonetheless, GOS/FOS intervention has already demonstrated its ability to normalize 5-HT concentrations in the frontal cortex and hippocampus [21], leading to behavioral improvements.

In the present study, *in utero* VPA-exposed mice exhibited an imbalance between Th17 and Treg cells in the spleen, consistent with observations in individuals with ASD [80]. In addition, *in utero* VPA exposure decreased the levels of HP, an acute-phase protein produced by the liver, in serum. Under healthy conditions, HP is increased by pro-inflammatory cytokines and has anti-inflammatory properties. The lack of HP allows for a stronger activation of dendritic cells, and therefore this can lead to a more potent activation and differentiation of T cells with a pro-inflammatory cytokines profile [81]. Conversely, HP levels have been shown to be increased in individuals with ASD [82]. Although the mechanisms underlying the decrease in HP levels following *in utero* exposure to VPA in mice are yet to be fully elucidated, the decrease may be attributed to the chronic inflammation induced by in utero VPA exposure. However, GOS/FOS supplementation normalized circulating HP levels and Th17/Treg cell balance in in utero VPA-exposed mice, thus improving the derailed immune system associated with in utero VPA exposure and potentially mitigating the pro-inflammatory context peripherally and centrally, and contributing to improving ASD-like behavior.

The immunomodulatory properties of 5-HT have previously been well-established [83–86]. Our study revealed that prenatal exposure to VPA decreased the number of 5-HT^+^ in the ileum. This reduction is associated with an increase in the number of Th17 cells in the spleen. It is likely that the diminished 5-HT levels, caused by a decrease in the number of 5-HT-producing cells, contributed to a shift towards a Th17 phenotype, as previously reported [87]. Consequently, it is plausible that elevating 5-HT levels could restore Th17/Treg cell balance [87–90]. Remarkably, GOS/FOS rescued the number of 5-HT^+^ cells in the ileum, thereby restoring Th17/Treg cell balance. This finding highlights the interplay between T lymphocytes and 5-HT. Moreover, apart from its immunomodulatory role, 5-HT also plays a crucial role in regulating tight junctions between intestinal epithelial cells to maintain gut barrier integrity by increasing ZO-1 expression and its redistribution to the tight junction region of the epithelial cells [91]. Disruption of these tight junctions can lead to increased gut permeability and mucosal inflammation [92]. In this study, *in utero* VPA exposure not only decreased the number of 5-HT^+^ cells but also reduced the expression of ZO-1 protein in the ileum. This indicated an increased permeability of the ileum, which was subsequently restored by GOS/FOS supplementation. Including a functional assessment of intestinal permeability would provide a more comprehensive understanding of intestinal barrier functionality. Taken together, our findings demonstrate that a prebiotic diet can improve intestinal 5-HT levels and maintain gut barrier integrity, possibly contributing to the reduction of mucosal inflammation.

As our study targeted microbes, we investigated the effect of GOS/FOS supplementation on the microbial composition of mice exposed to VPA during pregnancy. We found that regardless of exposure to PBS or VPA, the prebiotic diet had a significant effect on the community and structure of the gut microbiome. The prebiotic mix noticeably reduced alpha diversity indices. Additionally, the diet explained 24% of the observed variance between groups, consistent with previous research on prebiotics [93, 94]. We also observed a shift in B/F ratio only in VPA GOS/FOS mice. This indicates that GOS/FOS can influence alterations in gut microbial structure induced by *in utero* VPA exposure. Using LEfSe analysis, we identified 13 differentially abundant bacteria, when taking into account the lowest taxonomic rank, in mice exposed to PBS or VPA during pregnancy. Remarkably, five of the six bacteria associated with the PBS group were restored in VPA mice treated with GOS/FOS, including *Prevotella*. A previous study has reported a depletion of *Prevotella* in autistic children, which was increased after microbiota transfer therapy [12, 13]. Furthermore, we observed a positive correlation between SCFA (butyric acid and propionic acid) and key taxa found in control mice. Conversely, these SCFA showed a negative correlation with key taxa represented in *in utero* VPA-exposed mice. Moreover, we noticed a reverse pattern regarding branched chain fatty acids, suggesting a shift towards a proteolytic metabolic activity of the gut microbiome in mice with in utero VPA exposure, leading to adverse health effects [95]. Remarkably, GOS/FOS treatment prevented this shift. Altogether, our findings demonstrate that GOS/FOS diet restores key taxa profile and bacterial metabolic activity between mice exposed to PBS and VPA, ultimately improving ASD-like associated symptoms.

In summary, *in utero* exposure to VPA changes the gut microbiota composition, which is associated with a pro-inflammatory status, and ultimately disturbs social behavior and spatial memory. Our study demonstrated, for the first time, the therapeutic potential of GOS/FOS in reversing these changes by modulating the gut microbiota composition and immune system. This finding supports the pivotal role of the immune system in the gut-brain communication and the pathophysiology of ASD. Overall, our research provides valuable insights into the potential of dietary interventions to promote healthy gut microbiota and immune system function, and ultimately improve clinical outcomes for individuals with ASD.

### Supplementary information

Supplementary Figure 1

Supplementary figure legend
