# Supplementary figures and images for: Prebiotic diet normalizes aberrant immune and behavioral phenotypes in a mouse model of autism spectrum disorder
Source: Acta Pharmacol Sin. 2024 Apr 8;45(8):1591–603. doi: 10.1038/s41401-024-01268-x (PMC11272935; doi:10.1038/s41401-024-01268-x)

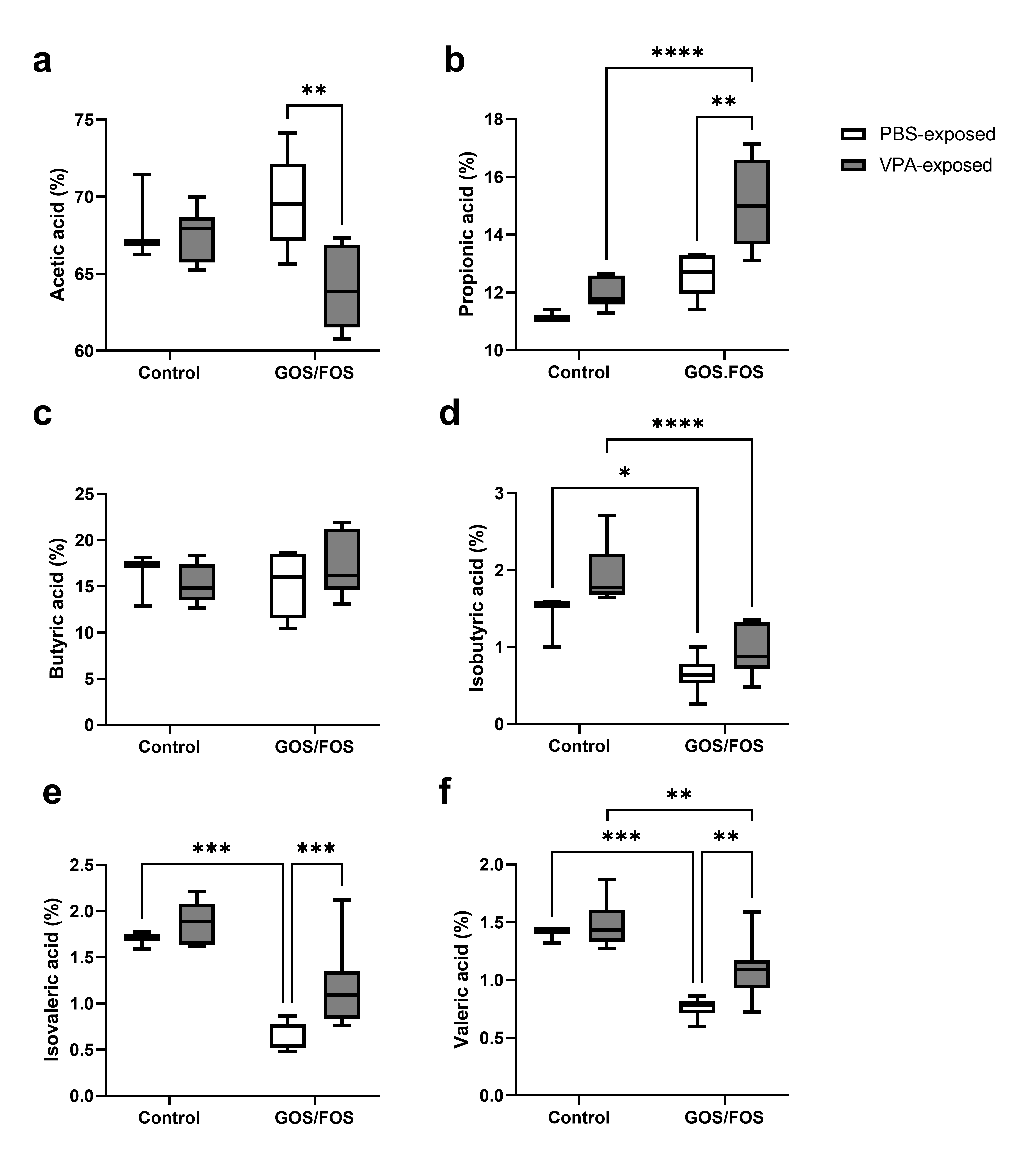

Supplement: Supplementary file 1 — Supplementary Figure 1 [file 41401_2024_1268_MOESM1_ESM.tif]
