# Supplementary material for: Prebiotic diet normalizes aberrant immune and behavioral phenotypes in a mouse model of autism spectrum disorder
Source: Acta Pharmacol Sin. 2024 Apr 8;45(8):1591–603. doi: 10.1038/s41401-024-01268-x (PMC11272935; doi:10.1038/s41401-024-01268-x)
Supplement: Supplementary file 2 — Supplementary figure legend [file 41401_2024_1268_MOESM2_ESM.docx]

**Supplementary Figure 1 Prebiotic diet changes SCFA production in PBS- and VPA-exposed mice.** Data represent SCFA percentages from cecal contents at P50. (**a**) Acetic acid, (**b**) propionic acid, (**c**) butyric acid, (**d**) isobutyric acid, (**e**) isovaleric acid, (**f**) valeric acid. ∗*P* < 0.05, ∗∗*P* < 0.01, ∗∗∗*P* < 0.001, ∗∗∗∗*P* < 0.0001. PBS control: *n* = 3 mice; VPA control: *n* = 6 mice; PBS GOS/FOS: *n* = 7 mice; VPA GOS/FOS: *n* = 9 mice.
